# 

**Published:** 1886-06-20

**Authors:** 


### TABLE OF CONTENTS.

ORIGINAL AND SELECTED ARTICLES.

A case of Hour-Glass Contraction of the Womb, with Retention of Placenta and followed by Internal Hemorrhage, by J. McF. Gaston, M.D., Professor of the Principles and Practice of Surgery in the Southern Medical College, Atlanta, Ga....205
Herniotomy, by C. J. Carter, M.D., of Georgia..207
Therapeutics and Neurological Therapeutics, by C. L. Dana, M.D....209
The Hygiene of Old Age, by H. C. Wood, M.D..214
Observations on the Treatment of Scarlet Fever....217
Clinical Notes on Antiseptics, by A. Mitchell, M.D., Los Angeles....219
The Panacea of Modern Gynecology....220

ABSTRACTS AND GLEANINGS.

American Medical Association....221
Saving Condemned Limbs....224
Tubercular Phthisis; Treatment of First Stage..226
Sulphate of Sodium....227
Belladonna as a Remedy in Acute Chronic Catarrh....228
Hyoscamine in Chorea; Insomnia in the Aged..229
Bromide in Diphtheria; Vaccinization....230
Convenience and Economy in the Use of Cocaine; The Pasteur Mission....231
Mag. Sulph. in Rheumatism; Foreign Body in the Nose; Drying up the Milk....232
On the Treatment of Ringworm; Endometritis....233
Remedies for Skin-Diseases in the Form of Spray; The Treatment of Varicocele; Treating Dysentery with Cocaine; Menthol in the Treatment of Urticaria and Pruritus; Parthenine in the Treatment of Facial Neuralgia....234

SCIENTIFIC ITEMS.

Inoculation as a Preservative against Consumption; Photographing Celestial Bodies...235
The Wonders of an Egg; Cattle Bones; The English "Parcels Post."....236

PRACTICAL NOTES AND FORMULÆ.

Useful Formulæ; Scarlet Fever....237

EDITORIALS AND MISCELLANEOUS.

Editorial Notices; The Atlanta Society of Medicine....238
A New Medical Society; The Texas Medical Association....239
Tribute to Dr. J. M. Johnson....240
In Memoriam....242
Receipted; Book Notice....243
Special Notices....244

